# Amperometric Monitoring of Dissolution of pH-Responsive EUDRAGIT^®^ Polymer Film Coatings

**DOI:** 10.3390/mi13030362

**Published:** 2022-02-25

**Authors:** Júlia Mestres, Francesca Leonardi, Klaus Mathwig

**Affiliations:** 1 Stichting Imec Nederland within OnePlanet Research Center, Bronland 10, 6708 WH Wageningen, The Netherlands; juliamestresmartinez@gmail.com; 2 School of Chemistry, University College Cork, Kane Building, T12 YN60 Cork, Ireland

**Keywords:** EUDRAGIT^®^, protective polymers, sensor protection, electroanalysis, fouling, polymer characterization

## Abstract

Electrochemical sensors are powerful tools for the detection and real-time monitoring of a wide variety of analytes. However, the long-term operation of Faradaic sensors in complex media is challenging due to fouling. The protection of the electrode surface during in vivo operation is a key element for improving the monitoring of analytes. Here, we study different EUDRAGIT^®^ controlled release acrylate copolymers for protecting electrode surfaces. The dissolution of these polymers—namely EUDRAGIT^®^ L 30 D-55 and EUDRAGIT^®^ FS 30 D—is triggered by a change in pH of the environment, and it is electrochemically monitored by detecting electrode access by means of a redox probe. The full dissolution of the polymer is achieved within 30 min and the electrode response indicates a complete recovery of the original electrochemical performance. We demonstrate that amperometric sensing is a practical and straightforward technique for real-time and in situ sensing of EUDRAGIT^®^ dissolution profiles. It will find future applications in determining the protection of polymer electrode coating in real matrices and in vivo applications.

## 1. Introduction

Real-time data are essential in many research fields ranging from monitoring of contaminants in rivers—allowing an early response in case of critical pollution events [1]—to the continuous monitoring of glucose—enabling better insulin dose decisions [2].

Real-time monitoring is challenging in the field of implantable and ingestible sensors [3,4]. Clinical assessment is traditionally performed using techniques involving delayed-time analysis (e.g., ELISA assays), or making use of invasive devices (e.g., catheters or biopsies). State-of-the-art approaches for sensing in the gastrointestinal (GI) tract are invasive and suffer from unreliability [5]. Real-time monitoring of analyte concentration profiles in the GI tract will provide valuable information about food fermentation and the effects of different diets as well as medical and nutritional supplements [6]. In addition, such a non-invasive in vivo methodology will be very valuable for monitoring regions of the body which are difficult to access with conventional methods, but challenging due to the continuous exposure to an aggressive and complex environment (aiming to digest everything) [7]. The gastrointestinal tract is an excellent example of an environment that would benefit from continuous and reliable monitoring for detecting specific biomarkers [8], but its exploration is still limited to invasive techniques and ex vivo laboratory assessment.

A common drawback affecting sensors exposed to biological fluids such as in the GI tract is the accumulation and adsorption of chemical and biological species (enzymes, hormones, proteins, bacteria, etc.) on the sensor surface [8]. The attachment of those molecules, i.e., biofouling, generates a drift in the sensor response, amplifies noise, lowers specificity and can even lead to failure of the sensor when a passivation layer is formed on the surface [4]. Electrochemical sensors are particularly susceptible to biofouling, as signal transduction takes place directly at the electrode surface.

To prevent biofouling effects, the protection of the electrode surface is a key element for long-term measurement. Here, we have employed a non-toxic stimuli-responsive acrylate co-polymer for the protection of the sensor surface. We investigated EUDRAGIT^®^ polymers, which are commercially used in pharmaceutical applications for time-controlled drug release [9] and conventionally characterized via common analytical techniques, i.e., UV spectrophotometry or HPLC-UV [10,11,12].

EUDRAGIT^®^ is stable in the acidic environment of the stomach and dissolves at the neutral pH in the intestine, showing a pH-controlled dissolution. For the purpose of sensor applications, this behavior can be exploited for the delayed activation of the sensor at a specific location in the GI tract. 

Ruiz-Valdepeñas Montiel et al. [13] employed cyclic voltammetry to evaluate a four-electrode sensor array protected by EUDRAGIT^®^ L100 polymer (soluble above pH 6), using different coating thicknesses. These sensors were used to measure glucose in untreated blood and saliva.

**Figure 1:**
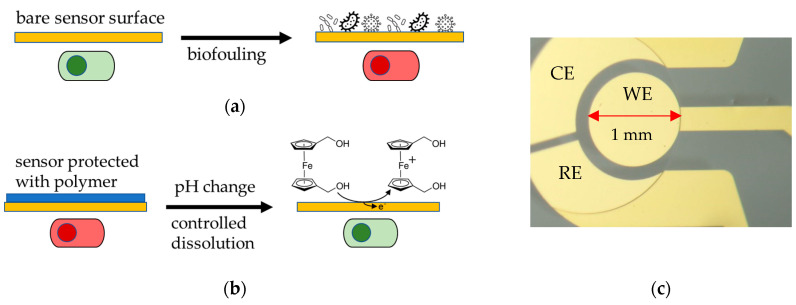
(**a**) Biofouling of an unprotected bare sensor surface hinders sensing due to non-specific adsorption on the surface (green: functional sensor, red: unfunctional sensor). (**b**) Experimental principle of this study: An EUDRAGIT^®^ polymer layer protects the Au electrode surface from biofouling; its dissolution is triggered by a change in pH. The dissolution process is continuously monitored electrochemically by biasing the electrode and observing an increase in Faradaic current as the surface becomes accessible for the oxidation of a Fc(MeOH)_2_ redox probe. (**c**) Micrograph of a gold working electrode (WE) with 1 mm diameter (on-chip quasi reference and counter electrode were not used and kept electrically floating during all experiments). The leading wire on the right side is insulated by an SU-8 resin.

Similarly, this study aims to characterize two pH-controlled release EUDRAGIT^®^ products, namely EUDRAGIT^®^ L 30 D-55 and EUDRAGIT^®^ FS 30 D, electrochemically. The protective polymer coating was applied prior to the sensor operation and dissolved once the sensor needs to be activated. The dissolution is triggered by the solution pH, and evaluated employing a 1,1′-Ferrocenedimethanol (Fc(MeOH)_2_) redox probe as target analyte. Once the coating dissolves, Fc(MeOH)_2_ can access the electrode surface by diffusion. We determined the active surface area, and thus, the degree of EUDRAGIT^®^ polymer dissolution, as the oxidation current of the redox probe by using voltammetry and amperometry (Figure 1).

## 2. Materials and Methods

### 2.1. Materials

EUDRAGIT^®^ FS 30 D, EUDRAGIT^®^ L 30 D-55 and PlasACRYL™ excipients were provided by Evonik Industries. Solutions were prepared in DI water using K_2_HPO_4_, KH_2_PO_4_ and 1,1’-Ferrocenedimethanol (97%) purchased from Sigma Aldrich and used without further purification. Fc(MeOH)_2_ is a well-known redox probe with a reversible electrochemical behavior. H_2_SO_4_ (1 M, product no. 1.60313) was purchased from Sigma Aldrich and used for cleaning the gold electrode.

### 2.2. Membrane Preparation

This work followed the preparation procedure and polymer formulation (Table 1) as recommended by Evonik [14].

EUDRAGIT^®^ FS 30 D was provided as an aqueous dispersion with 30% dry substance; it was first mixed with the corresponding amount of water and stirred for 10 min at 1000 rpm using a magnetic stirrer. Then, the mixture was added to the PlasACRYL™ T20 excipient, and the final suspension was stirred for 10 min at 1000 rpm. PlasACRYL™ T20 is a mixture containing 20% of mono and di-glycerides (GMS) and a plasticizer (triethyl citrate) and, due to its thixotropic behavior, must be shaken vigorously before use.

In an analogue manner, PlasACRYL™ HTP20—a ready-to-use thixotropic emulsion composed of 20% hydroxypropyl methylcellulose HPMC and triethyl citrate—was shaken and mixed with water for 10 min at 1000 rpm. This mixture was then added to the EUDRAGIT^®^ L 30 D-55 (provided as an aqueous dispersion with 30% dry substance) and stirred for 10 min at 1000 rpm.

After preparation, the polymers were stored refrigerated for a maximum of 3 months. EUDRAGIT^®^ coatings were deposited on a 1 mm electrode surface (see below) by drop-casting, using a METCAL DX-350 dispenser at a pressure of 2 bar in time-controlled mode (6 milliseconds), using a syringe of 3cc (Fisnar Europe QuantX) and a TE needle 27 GA 1”/2” clear (METCAL). After deposition, the coating was left to dry for 24 h at room temperature. Here, we exclusively studied coatings of a single deposited layer.

### 2.3. Methods

We used a PalmSense4 potentiostat controlled by PStrace v.8.5 software. This software was also employed for the analysis of Electrical Impedance Spectroscopy data. Electrochemical experiments were conducted in an electrochemical ‘All-in-One’ cell obtained from Micrux, using a 3-electrode configuration in unstirred conditions. The WE consist of a planar circular gold electrode (Micrux; ED-SE1-Au) with a diameter of 1 mm (inter-electrode relative standard deviation of 6% according to the fabricant). A Pt wire (BASInc; C1A/B MW-4130) and an Ag/AgCl (Microelectrode.inc; MI-402 flexible reference electrode) were used as a counter (CE) and reference electrode (RE), respectively (on-chip RE and CE were not used and kept electrically floating). Before the polymer deposition, the gold electrode was electropolished by performing a 12-scan CV routine at a scan rate of 0.1 V/s in a voltage window from -1 V to 1.3 V in 0.05 M H_2_SO_4_.

Cyclic voltammetry (CV) was performed within a voltage window ranging from 0 V to 0.5 V at scan rate of 50 mV/s at room temperature. Chronoamperometry (CA) measurements were conducted at 0.5 V for a 1 h duration experiment with a sampling time of 2 s at 37 °C. Finally, Electrochemical Impedance Spectroscopy (EIS) was performed in a frequency range of 10^5^ Hz to 10^−1^ Hz by superimposing an AC voltage of 10 mV to a DC voltage of 0.3 V.

The solution pH was controlled with phosphate buffer solution (PBS) at a concentration of 0.1 M. The target analyte (redox probe) consisted of 1 mM Fc(MeOH)_2_.

Current data are presented as the average ± standard deviation of a series of replicates. The percentage of a measured signal was determined by dividing the anodic peak current (*i*_pa_) of Fc(MeOH)_2_ oxidation by the *i*_pa_ that was recorded using the bare uncoated electrode.

## 3. Results and Discussion

### Dissolution Profile Characterisation

**Figure 2:**
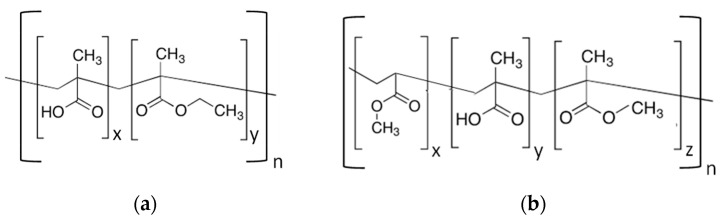
Chemical structure of (**a**) EUDRAGIT^®^ L 30 D-55 and (**b**) EUDRAGIT^®^ FS 30 D.

EUDRAGIT^®^ L 30 D-55 and FS 30D anionic copolymers possess the property of being soluble in water above a certain pH threshold (pH 5.5 and 7.0 respectively) [9]. As shown in Figure 2, EUDRAGIT^®^ L 30 D-55 is a methacrylic acid (MA) and ethyl acrylate copolymer (EA) (1:1 ratio of free carboxyl groups and ester groups), whereas EUDRAGIT^®^ FS 30D is a copolymer based on methyl acrylate, methacrylic acid (MA) and methyl methacrylate (MMA) (1:10 ratio of free carboxyl groups and ester groups).

**Figure 3:**
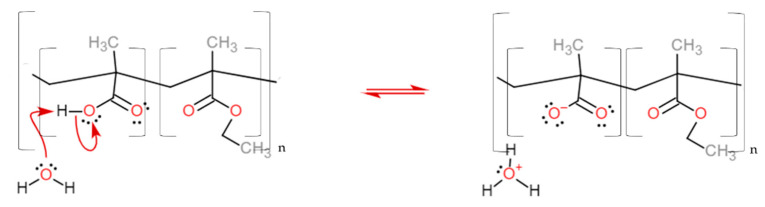
The reaction of the carboxylic acid monomer of the EUDRAGIT^®^ L 30 D-55 polymer with water leads to the polymer dissolution. The same reaction occurs for EUDRAGIT^®^ FS 30 D.

The dissolution mechanism starts with the diffusion of water into the polymer coating, forming a gel layer. The dissolution pH of these classes of polymers is tuned according to the methacrylic acid units along the chain [15,16]. Their deprotonation increases the solubility of the coating, which progressively diffuses towards the bulk of the solution (Figure 3, see also Video S1 in the Supplementary Materials).

The polymers L 30 D-55 and FS 30D were evaluated at different pH conditions to assess their dissolution profile above pH 5.5 and 7.0, respectively. This assessment aimed to verify the WE response towards a redox analyte in presence of the protective coating, as well as after its dissolution at a proper pH when the electrode surface becomes again accessible to the analyte.

In a first assessment, the two EUDRAGIT^®^ coatings were tested by cyclic voltammetry at room temperature (RT) under three different pH conditions: 0.5 pH units below the dissolution pH, at the dissolution pH, and 0.5 pH units above the dissolution pH. The increase in pH affects the stability of the film. The three experimental conditions were evaluated by observing the oxidation peak current (*i*_pa_) obtained from CV scans taken every 10 min (see Figure S1). The reference *i*_pa_ value was obtained from a bare WE and its value was 0.88 ± 0.04 µA (*n* = 6) at room temperature.

The results show how the coating behaves differently in the three regimes. When working below the critical dissolution pH, the coating is stable and current values barely increase. At the dissolution pH and above, the cathodic/anodic signal (*i_pc_*/*i_pa_*) of the redox probe appears, showing a reversible behavior as the coating dissolves (see Figure S2).

**Figure 4:**
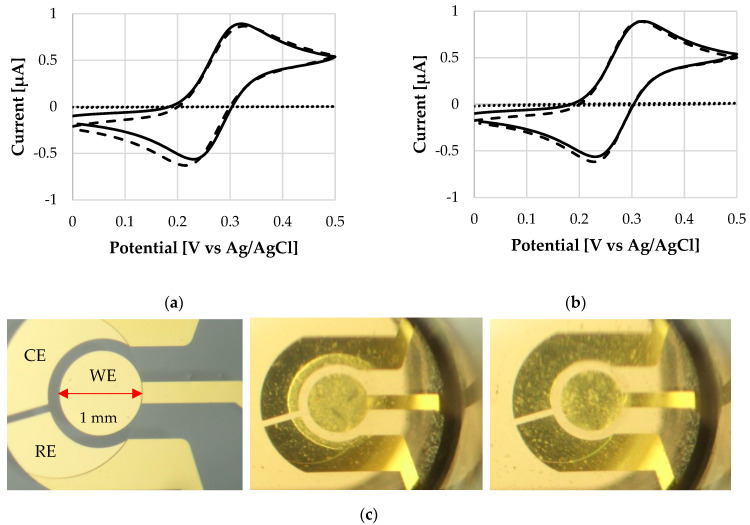
Cyclic voltammograms of the bare WE (plane line), and WE after the application of a polymeric coating (dotted line) and after polymer dissolution (dashed line) at 25 °C, of (**a**) L 30 D-55 polymer and (**b**) FS 30 D polymer. (**c**) From left to right: Micrograph of WE surface without any coating, WE with a coating on top at the beginning of its dissolution, WE after dissolution of the coating. Notice that the bulk solution becomes cloudy, see dissolution process in Video S1.

Figure 4 compares cyclic voltammograms before and after coating deposition, and after the dissolution. The oxidation peak values (*i*_pa_) amount to 0.87 µA and 0.90 µA for L30 D-55 and FS 30 D, respectively. CV traces clearly show that the electrochemical response is barely affected after the dissolution of the coating: no potential or current drift of the *i*_pa_ peaks occurs. The same scenario is observed for *i_pc_* peaks with current values of -0.79 µA and -0.86 µA for L 30 D-55 and FS 30 D, respectively. The reference *i*_pc_ of a bare electrode is -0.80 ± 0.04 µA (*n* = 6). This first investigation indicates that the polymer does not damage the sensor surface. Figure 4 also shows the behavior of the electrode coated with the polymeric thin film immediately after the exposure to the solution. The low signal trace (dotted line, current values < 0.01 µA) indicates a high resistance, as the dissolution process is still at a very early stage.

Electrochemical Impedance Spectroscopy (EIS) was used as additional characterization for studying the blocking behavior of the thin film. The EIS behavior was firstly investigated before the application of the coating, secondly using the electrode coated with the protective polymer and finally after the exposure of the coating to the buffer solution at a dissolution pH (see Table S1). Measurements were performed in a faradic regime using a solution of PBS 0.1 M containing 1 mM Fc(MeOH)_2_ as a redox probe (see Methods Section 2.3). The pH value was kept at 6.0 and 7.5 for polymers L 30 D-55 and FS 30 D, respectively. 

The response was interpreted using an equivalent circuit, i.e., a Randles cell circuit [17,18]. A simplified Randles cell circuit describes the process on an electrode surface that follows the Helmholtz double-layer model [19]. We employed this circuit model since the main aim of the experiment was to evaluate the gold surface before and after the coating. To investigate the EIS property of the electrode covered with the polymeric film, we immersed the substrate and performed an EIS investigation at the early beginning of the immersion (t = 0), when the film is still present on the sensor surface. The next recording was taken after 60 min, following complete dissolution of the polymeric coating. 

Prior the coating deposition, the WE displayed an ideal metal behavior with a time constant (R_ct_/C_dl_) appearing at high frequencies, while the lower frequency region was dominated by diffusion (Z_w_) (see Table S1). Our fitting resulted in a C_dl_ value in agreement with capacitance values of standard gold electrodes immersed in an electrolyte (16 µF/cm^2^). This capacitance behavior also appears in the Bode plot, with a phase angle of almost -90 degrees in a frequency range of 1000 Hz to 700 Hz. The diffusion of the redox probe in one dimension (Warburg impedance Z_w_) can be observed in the Nyquist Plot as a diagonal line with a slope of 45° (or at the phase angle plot as a −45° value). 

The previous behavior disappears when the polymeric coating is deposited (t = 0); the film increases the resistive behavior of the working electrode resulting in an EIS response of poor consistency (see Figure S3).

Once the coating was dissolved, the original behavior was restored. This is evidenced by the almost identical EIS response showing circuit parameters that are comparable to the pristine WE. The identical C_dl_ and the slight variation of the R_ct_ confirms the results obtained by cyclic voltammetry.

**Figure 5:**
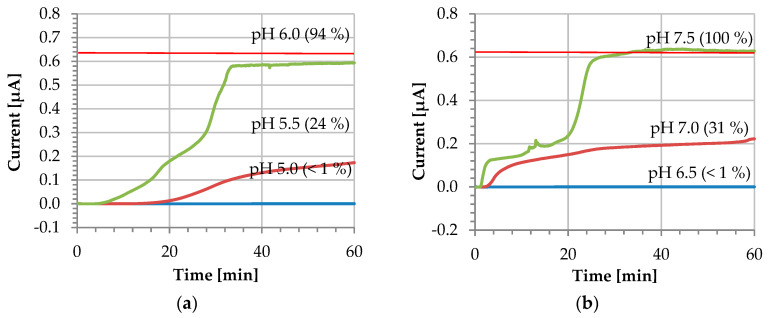
Dissolution profile at different pH values (i vs. t) of: (**a**) L 30 D-55 and (**b**) FS 30 D polymers. The red horizontal line indicates the current value of a bare gold electrode 0.64 ± 0.02 µA (*n* = 7).

For in vivo sensing applications in bodily fluids, sensors must perform at 37 °C. In continuous monitoring, sensors are often operating in chronoamperometric mode, which we have chosen to carry out our experiments [20]. In these experiments, the coating was initially applied on the electrode surface and allowed to dry for 24 h at room temperature. The electrochemical setup was then heated to 37 °C and the CA was run for 1 h with an applied bias of 0.5 V. As a monitoring solution, a 0.1 M PBS containing 1 mM Fc(MeOH)_2_ as redox probe was employed. Figure 5 shows profiles obtained at different pH values. The current value obtained from a clean bare gold WE amounts to 0.64 ± 0.02 µA (*n* = 7).

When the electrode surface is completely covered and the pH is kept below the polymer dissolution pH, the current does not increase—instead, it remains at a residual level of approximately 0.4 nA. The blue line in Figure 5 corresponds to this scenario and demonstrates the stability of the coating during the entire recording duration of 60 min. The coated sensor was further tested at the exact polymer dissolution pH, where a slight increase of the current is observed. In this case, the dissolution remains incomplete and the current increases to 24% and 31% of the pristine reference value after 60 min for L 30 D-55 and FS 30 D, respectively. According to the manufacturer’s information [14], the higher the pH is raised above the dissolution pH, the faster the dissolution rate will be. For our experimental conditions, the complete dissolution of the coating is observed for the exact polymer dissolution pH resulting in a very long activation time not practical for real-world applications. 

Finally, when the solution employed is 0.5 pH units above the polymer dissolution pH, the coating dissolves completely. This can be observed in Figure 5 as an increase of the current stabilizing after 30 min, with values of 0.59 ± 0.01 µA and 0.63 ± 0.01 µA for L30 D-55 and FS 30 D, respectively. At this point, the pristine electrode conditions are fully restored, and the coating is fully dissolved. For both polymers, the coating dissolves repeatedly after approximately 30 min at a pH of 0.5 pH units above the dissolution pH (see also Figure S1 in the Supplementary Material). We attribute different shapes of the *I-t* dissolution curve to varying uncontrolled mass transport (unstirred solution). 

## 4. Conclusions

EUDRAGIT^®^ L 30 D-55 and FS 30 D copolymers were studied using electrochemical techniques. These two copolymers were deposited as thin films and their dissolution behavior was investigated by changing the pH. By comparing cyclic voltammograms before and after the polymer application we confirm that the polymeric coating does not damage the surface of the gold electrode, effectively protecting the sensor surface until the proper pH dissolves the coating. The EIS data confirm this conclusion, by providing consistent values of the Randles circuit parameters before and after the dissolution of the coating.

Chronoamperometry provides a continuous characterization of the copolymers’ dissolution profiles. Below the dissolution pH of the coating, the electrode surface is effectively isolated from the bulk solution (current < 1% signal) at 37 °C. A gradual exposure of the WE surface is observed when the pH is raised above the coating dissolution pH, with a total recovery of the electrode signal after 25 to 30 min. When working with these polymers, it is important to consider that the dissolution rate is affected by parameters such as coating thickness or the number of applied polymer layers [13].

Electrochemical techniques have been proven to be an appropriate tool for the characterization of the dissolution profile of EUDRAGIT^®^ acrylate copolymers. Drop-casting the polymer precursor on top of a substrate is a fast and easy method that can be upscaled and tuned according to the application and the desired pH of dissolution.

This type of copolymer can be used as a practical method for the protection of sensors that require a time-controlled activation at a specific pH. In sensing applications, the optimization of the coating formulation improves the stability of the sensor and prevents fouling degradation of the surface when real-time monitoring in a harsh environment is required. In addition, this method allows tailoring the activation time of the sensor by protecting its surface until the target site is reached. Future work will aim to study the behavior on different sensor surfaces and the reproducibility in complex matrices.

## Figures and Tables

**Table 1:** EUDRAGIT^®^ polymer formulation.

**Function**	**Ingredient**	**[%]**
Polymer	EUDRAGIT^®^ FS 30 D	60.6
Anti-tacking	PlasACRYL™ T20	9.1
Diluent	Water	30.3
**Function**	**Ingredient**	**[%]**
Polymer	EUDRAGIT^®^ L 30 D-55	57.0
Anti-tacking	PlasACRYL™ HTP20	14.6
Diluent	Water	28.5

## Data Availability

The data presented in this study are available on request from the corresponding authors.

## Supplementary Materials

The following are available online at https://www.mdpi.com/article/10.3390/mi13030362/s1, Figure S1: Dissolution profile at different pH values at RT using the oxidation peak values (*i_pa_*) versus time (h), Figure S2: Cyclic voltammograms taken every 10 min of a WE covered by the two polymers at RT, Table S1: EIS data of the fitted Randles circuit before the application of the coating and after its dissolution, Figure S3: Nyquist and Bode plots obtained by measuring the WE coated at t = 0 h, Video S1: Dissolution of an EUDRAGIT^®^ L 30 D-55 coating (sped up 12 times).
