# Supplementary material for: Amperometric Monitoring of Dissolution of pH-Responsive EUDRAGIT® Polymer Film Coatings
Source: Micromachines (Basel). 2022 Feb 25;13(3):362. doi: 10.3390/mi13030362 (PMC8949041; doi:10.3390/mi13030362)
Supplement: Supplementary file 1 [file micromachines-13-00362-s001.zip › Supplementary information_Amperometric monitoring of dissolution of ph responsive EUDRAGIT polymer film coatings.pdf]

Supplementary Material for:

Amperometric monitoring of dissolution of pH-responsive EUDRAGIT® polymer film coatings

Júlia Mestres-Martínez,[1,2] Francesca Leonardi[1] and Klaus Mathwig[1]

[1] *Stichting imec Nederland within OnePlanet Research Center, Bronland 10, 6708 WH Wageningen, The Netherlands*
[2] *School of Chemistry, University College Cork, Kane Building, T12 YN60, Cork,*

Email: francesca.leonardi@imec.nl, klaus.mathwig@imec.nl

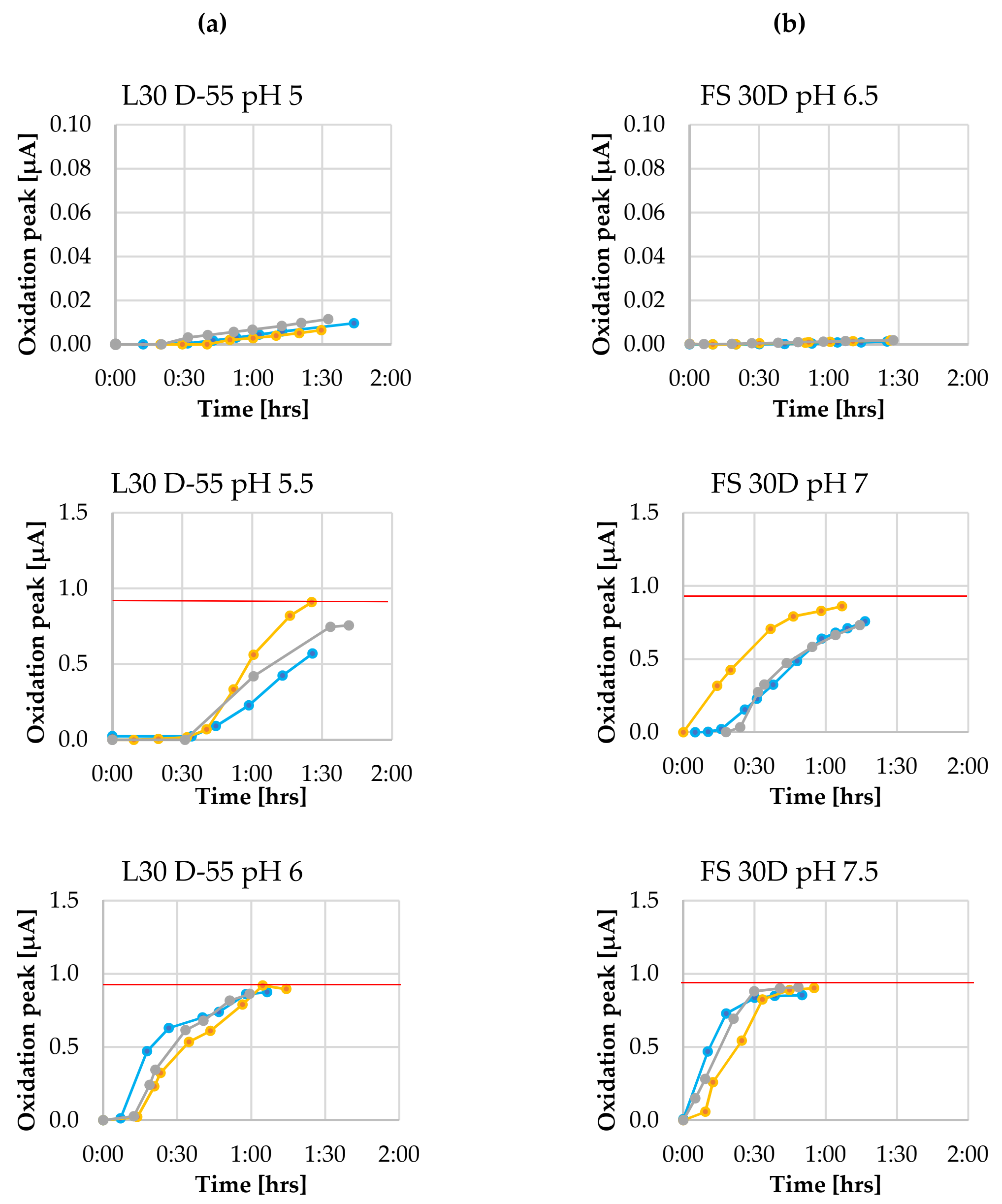

Figure S1. Dissolution profiles at different pH values at RT using the oxidation peak values (i_{pa}) *versus* time (hrs). (a) EUDRAGIT® L30 D-55 and (b) EUDRAGIT® FS 30D.

Blue, grey, and orange values represent 3 different replicates (n = 3). The horizontal plane red line (-) indicates the oxidation peak of the bare electrode: 0.88 ± 0.04 µA (n = 6).

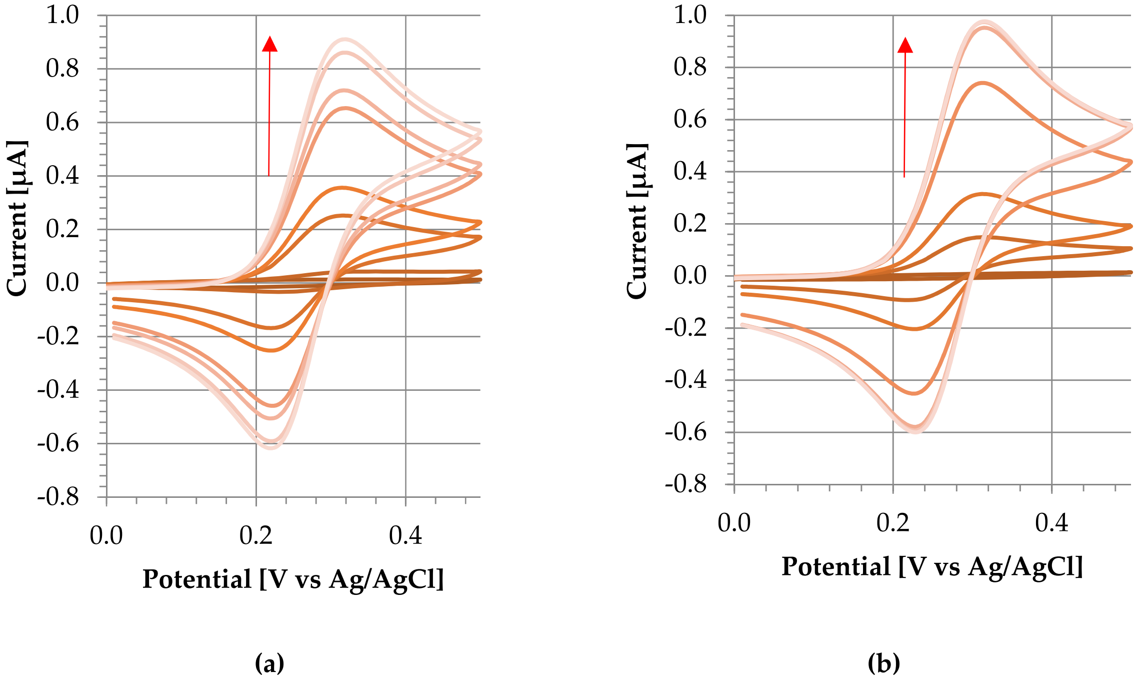

Figure S2. Cyclic voltammograms taken every 10 minutes of a WE covered by the two polymers at RT, scan rate of 50 mV/s. The scans are displayed from darker (t = 0 hrs) to lighter (t = 1 hrs). (a) L30 D-55 exposed to a pH 6 solution and (b) FS 30D exposed to a pH 7.5 solution.

Table S1 (on following page). EIS data of the fitted Randles circuit before the application of the coating and after its dissolution. Randles circuit used for EIS fitting, where R_s = solution resistance; R_{ct} = charge transfer resistance (related to the kinetics of heterogeneous charge transfer, also called polarization resistance); Z_w = Warburg impedance (diffusion process); C_{dl} = double layer capacitance.

For both plots, the plane red line (-) corresponds to the circuit fitting. In the Nyquist plot the dotted line (●) indicates the measured values. In the Bode plot, the dotted line (●) indicates the measured phase *versus* the frequency, while triangles (▲) the measured |Z| *versus* the frequency.

Circuit element	L30 D-55 before the coating	L30 D-55 after the coating	FS 30D before the coating	FS 30D after the coating
R_s [Ω]	636.3	695.4	702.8	696.5
R_{ct} [Ω]	8.18E+4	1.38E+5	7.69E+4	1.88E+4
C_{dl} [F]	1.28E-8	1.69E-8	1.77E-8	4.43E-8
Z_w [Ω $s^{-1/2}$]	1.28E+5	2.11E+5	1.56E+5	1.27E+5
$\sqrt{\chi^2}$ [%]	15	25	14	9

Nyquist plot ((-Z″ vs Z′))

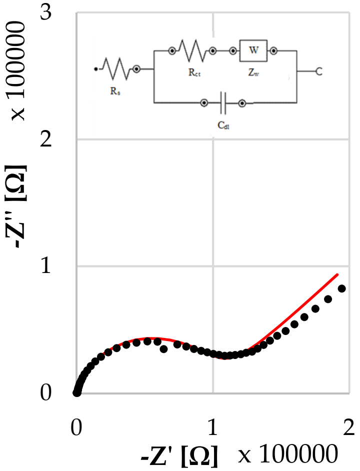

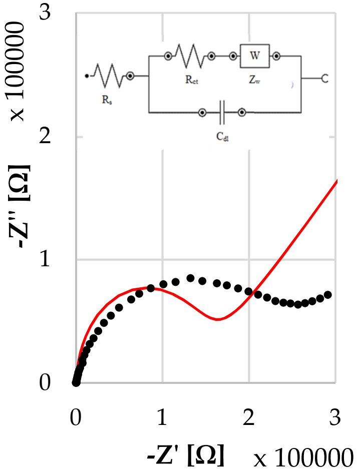

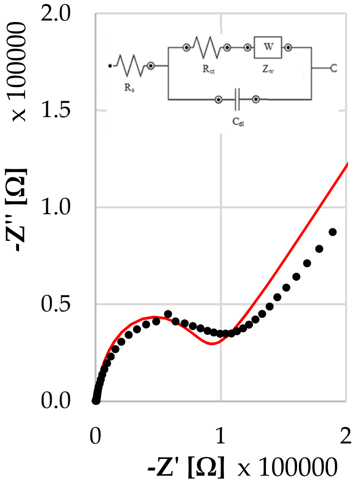

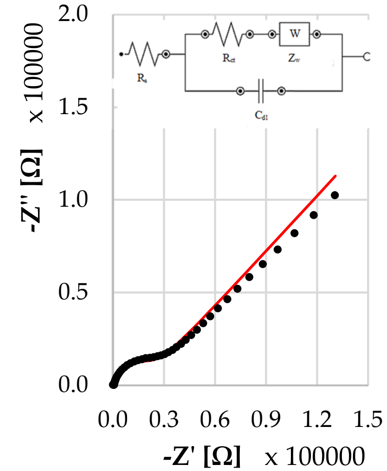

Bode plot (- Phase and |Z| vs log10(Hz))

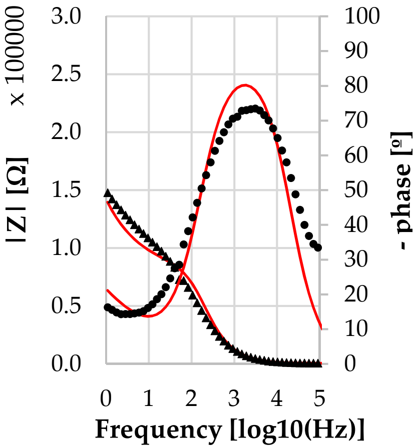

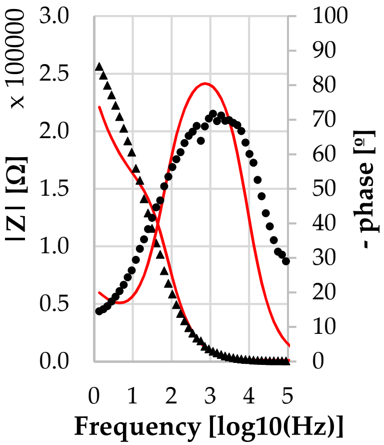

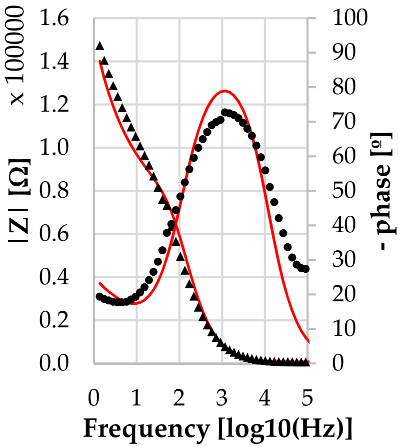

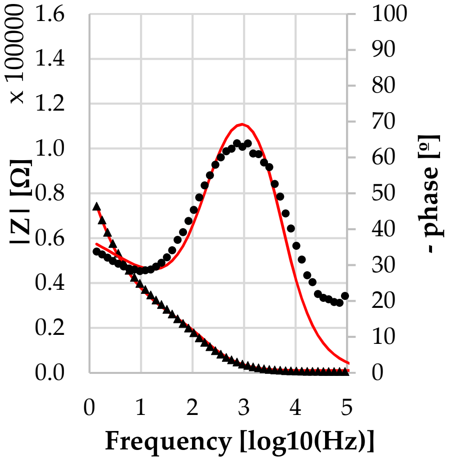

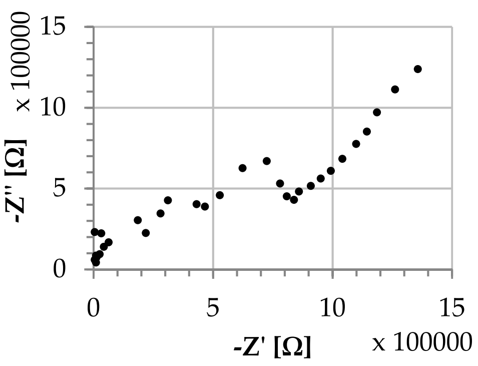

(a)

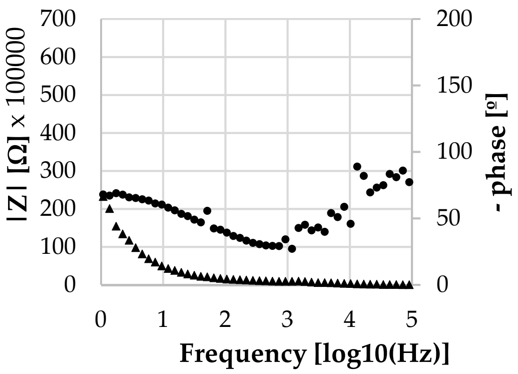

(b)

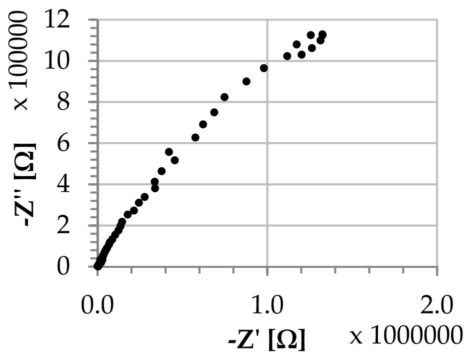

(c)

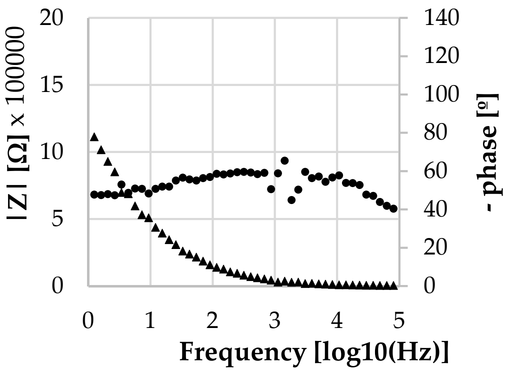

(d)

Figure S3. Nyquist and Bode plots obtained by measuring the WE coated at t = 0 hrs. The resistivity produced by the thin layer does not allow to acquire any quantitative data. In the Nyquist plot the dotted line (●) indicates the measured values. In the Bode plot, the dotted line (●) indicates the measured phase *versus* the frequency, while triangles (▲) the measured |Z| *versus* the frequency. (a, b) L30 D-55. (c, d) FS 30D.

Video S1. Dissolution at pH 6 and room temperature (RT) of an EUDRAGIT® L30 D-55 coating (speed up 12 times), drop-casted on top of a Micrux gold electrode.

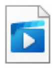

Video S1.MP4
